# Genotype-phenotype correlations of TGFBI p.Leu509Pro, p.Leu509Arg, p.Val613Gly, and the allelic association of p.Met502Val-p.Arg555Gln mutations

**Published:** 2011-05-05

**Authors:** Florence Niel-Butschi, Bernadette Kantelip, Justyna Iwaszkiewicz, Vincent Zoete, Mathieu Boimard, Marc Delpech, Jean-Louis Bourges, Gilles Renard, François D’Hermies, Pierre-Jean Pisella, Christian Hamel, Bernard Delbosc, Sophie Valleix

**Affiliations:** 1 Inserm, U1016, Institut Cochin, CNRS, UMR 8104, Université Paris-Descartes, Paris, France; 2 Service d’Anatomie Pathologique, CHU de Besançon, France; 3 Molecular Modelling Group, Swiss Institute of Bioinformatics, Lausanne, Switzerland; 4 Laboratoire de Biochimie et Génétique Moléculaire de l’hôpital Cochin, Assistance Publique Hôpitaux de Paris, Franc; 5 Service d’Ophtalmologie, Hôpital Hôtel-Dieu, Université Paris-Descartes, Assistance Publique Hôpitaux de Paris, France; 6 Service d’Anatomie Pathologique, Hôpital Hôtel-Dieu, Paris, France; 7 Service d’Ophtalmologie, Hôpital de Tours, France; 8 Centre des affections sensorielles rares, Montpellier; 9 Service d’Ophtalmologie, CHU de Besançon, France

## Abstract

**Purpose:**

Investigate the genotype-phenotype correlations for five *TGFBI* (transforming growth factor, beta-induced) mutations including one novel pathogenic variant and one complex allele affecting the fourth FAS1 domain of keratoepithelin, and their potential effects on the protein’s structure.

**Methods:**

Three unrelated families were clinically diagnosed with lattice corneal dystrophy (CD) and one with an unclassified CD of Bowman’s layer. Mutations in the *TGFBI* gene were detected by direct sequencing, and the functional impact of each variant was predicted using in silico algorithms. Corneal phenotypes, including histological examinations, were compared with the literature data. Furthermore, molecular modeling studies of these mutations were performed.

**Results:**

Two distinct missense mutations affecting the same residue at position 509 of keratoepithelin: p.Leu509Pro (c.1526T>C) and p.Leu509Arg (c.1526T>G) were found to be associated with a lattice-type CD. The novel p.Val613Gly (c.1828T>G) *TGFBI* mutation was found in a sporadic case of an Algerian individual affected by lattice CD. Finally, the Bowman’s layer CD was linked to the association in cis of the p.Met502Val and p.Arg555Gln variants, leading to the reclassification of this CD as atypical Thiel-Behnke CD. Structural modeling of these *TGFBI* mutations argues in favor of these mutations being responsible for instability and/or incorrect folding of keratoepithelin, predictions that are compatible with the clinical diagnoses.

**Conclusions:**

Description of a novel *TGFBI* mutation and a complex *TGFBI* allele further extends the mutational spectrum of *TGFBI*. Moreover, we show convincing evidence that *TGFBI* mutations affecting Leu509 are linked to the lattice phenotype in two unrelated French families, contrasting with findings previously reported. The p.Leu509Pro was reported to be associated with both amyloid and non-amyloid aggregates, whereas p.Leu509Arg has been described as being responsible for Epithelial Basement Membrane Dystrophy (EBMD).

## Introduction

Several clinical forms of autosomal dominant corneal dystrophies (CDs) are caused by missense mutations in the human transforming growth factor β-induced gene (*TGFBI*; OMIM *601692) that codes an ubiquitous 68 kDa extracellular matrix protein named keratoepithelin (KE) [1,2]. Depending on the *TGFBI* mutation, the entire mutated KE, or its proteolytic fragments, accumulate in the corneal stroma with distinct ultrastructures forming amyloid (Lattice Corneal Dystrophies; LCD), rod-shaped crystalloid structures (Granular CD type 1 and Reis-Bucklers CD), a combination of amyloid and rod-shaped bodies (Granular CD type 2), or “curly fibers” (Thiel-Behnke CD) [3,4]. The accumulation of KE aggregates in the different CD forms occurs at different locations within the corneal stroma and leads to corneal clouding and visual impairment with varying degrees severity. The current treatments for *TGFBI* corneal dystrophies are penetrating keratoplasty or excimer laser phototherapeutic keratectomy, both of which usually restore vision in affected patients [3,5]. Until now, more than 40 *TGFBI* mutations have been described in many different countries, and have been shown to affect either one of the two mutational hot spot residues Arg124 and Arg555, or to be clustered in the fourth fasciculin-like domain 4 (FAS1–4) of KE corresponding to amino acids 502–632 [3,6]. All genetic analysis worldwide has highlighted a remarkable genotype-phenotype correlation, allowing a better clinical-molecular classification of this group of diseases. Now, while the insight offered by these findings remains valuable, the genotype-phenotype correlation of these corneal dystrophies has gradually gained in complexity; it has been well demonstrated that some *TGFBI* mutations have shown significant phenotypic variability.

The precise function of keratoepithelin is still not elucidated, but this protein contains four homologous tandem repeat domains of 140 amino acids known as FAS1 domains, which are similar to those found in fasciculin I, a *Drosophilia* neuronal adhesion molecule [7]. FAS1 domains are known to mediate cell adhesion and migration by interacting with diverse matrix proteins such as fibronectin, collagen, laminin, proteoglycans, and also with specific integrins [8-10]. The KE protein fourth FAS1 domain’s structure is available in the protein data bank (PDB). The NMR (Nuclear Magnetic Resonance) structures are stored under 1X3B PDB ID and a recently released crystal structure under 2VXP PDB ID. There are no significant differences between the structures determined using both methods. The structure of keratoepithelin consists of a seven-stranded β wedge and several α helices in a novel arrangement, which bears no similarities to any other domain of known structures [7,11].

In this study, we report the phenotype of four distinct *TGFBI* mutations affecting the fourth FAS1 domain. They include a novel *TGFBI* mutation, p.Val613Gly, the allelic association of p.Arg555Gln and p.Met502Val mutations, and two *TGFBI* variants affecting the same residue, p.Leu509Pro and p.Leu509Arg. The p.Leu509Pro variant has previously been reported in one German family with a geographic pattern-like clinical phenotype associated with both hyaline and amyloid stromal deposits. In contrast, the p.Leu509Arg variant was detected in one French family and diagnosed as an Epithelial Basement Membrane Dystrophy (EBMD) [12,13]. In our two, four-generation French families with autosomal dominant dystrophy, we found that Leu509 variants both segregated, with geographic opacities seen at the level of Bowman’s layer and with lattice lines in the deeper layers of the corneal stroma, consistent with a diagnosis of lattice-type corneal dystrophy. Histologic examinations of diseased p.Leu509Pro corneas in our French patients demonstrated that all deposits were amyloid, in contrast to the two types reported for the German family, being amyloid and granular [13]. This report therefore provides further evidence of ongoing complexities concerning the pathogenesis and classification of *TGFBI* corneal dystrophies. Finally, our structural modeling allows us to present the predicted effects of the four different *TGFBI* mutations outlined above, providing insight into their disease mechanisms.

## Methods

### Patients

We analyzed three families (A, B, and C), one isolated case (case D), and a subset of four patients with EBMD. All subjects underwent clinical examinations (visual acuity, slit lamp biomicroscopy) in an ophthalmology unit and histopathologic records were reviewed on a case-by-case basis. The pedigree of each family was established and all were consistent with a dominant autosomal transmission of the corneal disease. All of the four rare or new *TGFBI* mutations (p.Leu509Pro, p.Leu509Arg, p.Val613Gly, and p.Met502Val) identified in the patients reported here have been screened in control subjects using direct sequencing of the corresponding *TGFBI* exons to determine whether they were polymorphims or disease-mutations. The control population consisted of 100 unrelated French individuals who showed normal corneal appearance after slit-lamp biomicroscopy examination. Additionally, our study also enrolled more than 60 French families affected with *TGFBI* corneal dystrophies, which were sent to our laboratory for mutations screening. The Algerian control population included a set of 100 unrelated, unaffected individuals who, according to medical records, had no family history of corneal disease.

### Molecular analysis

Blood samples were collected from patients for DNA analysis. Written informed consent was obtained from all the individuals who participated in the study. Genomic DNA was extracted from peripheral blood lymphocytes using a standard phenol/chloroform extraction procedure. PCR amplification of exons 4 to 17 and flanking splice sites of the *TGFBI* gene was achieved (primers used are presented in Table 1), and all amplicons were directly sequenced on both strands according to the protocols accompanying the BigDye Terminator v3.0 Cycle Sequencing Kit (Applied Biosystem-Life Technologies, Courtaboeuf, France). An ABI PRISM^®^ 3130xl DNA Sequencer (Applied Biosystems) was used to collect the sequence data. Sequences were aligned and compared with the reference *TGFBI* genomic sequence (GenBank NM_000358.1) using the SeqScape software v2.5 (Applied Biosystems).

**Table 1:** Primer sequences used for amplification of *TGFBI*.

**Exon**	**Primer name**	**Primer sequence**	**Melting temperature**	**Product size (bp)**
4	TGFBI-4F	cctcgtcctctccacctgta	60	328
	TGFBI-4R	tcggggaagtaaggcagttc	61	
5	TGFBI-5F	gtctgcagcccctaactgac	60	317
	TGFBI-5R	cacaaagagggtgggttgtc	60	
6	TGFBI-6F	gcttgtggaacccacatttt	60	392
	TGFBI-6R	tcaggggaacctgctctatg	60	
7	TGFBI-7F	tgggtttggcttctgttttc	60	376
	TGFBI-7R	catggcaggtggtatgttca	60	
8	TGFBI-8F	gggtcctcatctgagagaacag	60	480
	TGFBI-8R	gtcacaacccacacatttgc	60	
9	TGFBI-9F	cgagatgacattcctgctga	60	369
	TGFBI-9R	ttttggttgagctgagtgga	59	
10	TGFBI-10F	tccaaactcaaggagggatg	60	365
	TGFBI-10R	tcagcaaccagttctcatgc	60	
11	TGFBI-11F	tgaccctgctacatgctctg	60	374
	TGFBI-11R	ccatcccaagtctggaaggt	61	
12	TGFBI-12F	cctctcagcgtggtgaggta	61	261
	TGFBI-12R	ggccctgagggatcactact	60	
13	TGFBI-13F	gggcagggagttcttcattt	60	351
	TGFBI-13R	gctgcaacttgaaggttgtg	60	
14	TGFBI-14F	ctgggcgacaagattgaaac	61	433
	TGFBI-14R	tgtggtgcattcaaaaccaa	61	
15	TGFBI-15F	ggaaatgtgagccagaaagc	60	284
	TGFBI-15R	agcagccaaggaagacagg	60	
16	TGFBI-16F	accttccccttcctcttcct	60	281
	TGFBI-16R	caaaggccaggcttctttta	60	
17	TGFBI-17F	ttggccctggtccttgag	62	250
	TGFBI-17R	gcggcccatgtacattaaa	59	

### Histologic examination

The available corneal buttons were bisected immediately after grafting. Each button was fixed in Bouin's solution and was processed for evaluation. Corneal sections were stained with hematoxylin and eosin, periodic acid-Schiff, Alcian blue, Masson's trichrome, red Congo, and thioflavinT.

### In silico analysis by SIFT, PolyPhen, SNPs3D, and PMut

Bioinformatics approaches were used to identify highly conserved areas of a gene through a multiple sequence alignment analysis across numerous species or related proteins, and thereafter, to predict the potential impact of a residue in addition to structural data. These programs take into account, to varying degrees, physico-chemical properties of proteins (a change in charge or in the hydrophobicity, or a helix-breaking propensity), and their three-dimensional structures. Different algorithms (SIFT, PolyPhen, SNPs3D, and PMut) were used to evaluate the possible functional impact of the keratoepithelin amino acid substitutions identified here.

### Molecular modeling

The protein structure used in the modeling part is the keratoepithelin protein fourth FAS1 domain NMR structure, which is deposited in the protein data bank (PDB) under code 1x3b. The structure with the most favorable ANOLEA score was selected from the 20 deposited was selected and used to perform the subsequent calculations [14]. The importance of the amino acid side chains in certain positions, and the influence of the mutations in these positions on the structural stability were studied using computational alanine scanning. The software used for this purpose was FoldX 2.5.1 [15].The procedure was as follows: in a protein model, residues of interest were mutated to alanine by replacing their side chains with methyl groups and the folding free energy, also referred to as the stability, of the resulting mutants was compared to that of the wild type protein. The folding free energy is the difference in free Gibbs energy between the folded and unfolded states for a given sequence. The result thus estimates the influence of a given residue side chain on the stability of the protein. To study the influence of Gly substitution on the Val613 position in greater detail, we performed a 10 ns-long molecular dynamics (MD) simulation of the wild type NMR structure and of the NMR structure with the Val613Gly mutation. The mutation was introduced using Chimera software [15]. The MD simulation was conducted in the CHARMM force field using the GROMACS molecular dynamics package (Department of Biophysical Chemistry, University of Groningen, The Netherlands) in a water box with periodic boundary conditions [16,17]. After a steepest descent minimization and 1 ns-long equilibration, the 10 ns-long simulation was performed at 300 K for both the wild type and p.Val613Gly structures. The folding free energies were calculated using the FoldX 2.5.1 program for 100 conformations regularly extracted from the MD simulation (one every 100 ps).

## Results

### Clinical phenotypes

#### Family A

**Figure 1:**
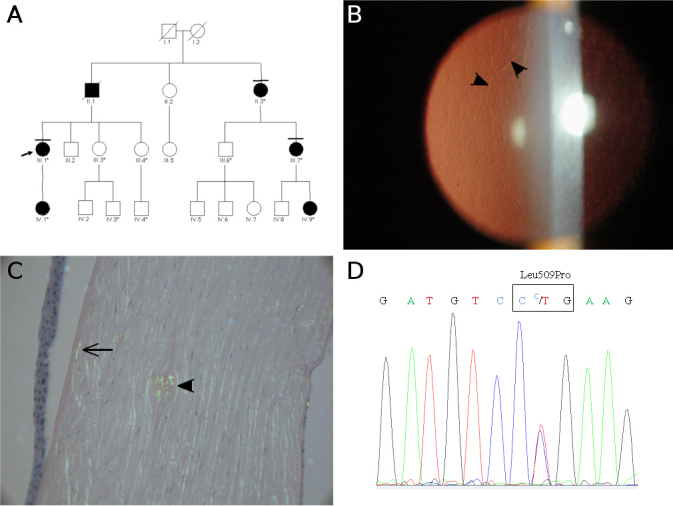
Family A. **A**: Pedigree showing a four-generation family affected by lattice-type corneal dystrophy. Open and closed symbols indicate unaffected and affected individuals, respectively, arrows indicate the proband in each family, and asterisks indicate members who were examined clinically and genetically. A bar on top of a symbol indicates that patients had received a bilateral grafted in both eyes. Diagonal lines indicate deceased individuals. **B**: Retroillumination slit-lamp view of patient IV-1 at 21 years of age. The left eye contained a network of thick lattice lines (arrowhead) and dots. **C**: Congo red positive deposits of amyloid aggregates are found in subepithelial (fine arrow) and mild corneal stroma (arrowhead). Green birefringence is visible with a polarizing ﬁlter. Stromal deposition of amyloid substance distorts the architecture of corneal lamellae. **D**: The electropherogram of exon 11 of the *TGFBI* gene is shown. The proband III.1 has a heterozygous thymine to cytosine change at codon 509 in exon 11 (c.1526T>C, p.Leu509Pro).

This family was clinically evaluated in the Department of Ophthalmology at Besançon Hospital. The proband was a French woman born in 1961 (individual III.1, Figure 1A), who was first examined at age 20 for a giant epithelial ulcer of the right cornea that was initially diagnosed as EBMD. However, slit-lamp examination revealed bilateral subepithelial opacities that appeared geographic in shape and that were combined with stromal fine lattice lines (Figure 1B). Over the ensuing years, this proband complained of recurrent episodes of epithelial erosions with pain and photophobia, and her visual acuity progressively declined because of corneal opacification. She underwent penetrating keratoplasty at age 33 on her right eye, and at age 36 on her left eye, and the corneal buttons were examined histopathologically. On histological examination, specimens from both eyes exhibited irregular epithelial thickening associated with focal interruption of the Bowman layer, secondary to the presence of amorphous eosinophilic linear deposits. Additional eosinophilic round aggregates, which were small in size, were observed randomly spread within the anterior and mild corneal stroma. These eosinophilic deposits did not stain red with Masson trichrome, but all stained orange with Congo red and displayed apple-green birefringence under polarized light, characteristic of amyloid aggregates (Figure 1C). The endothelium and Descemet’s membrane appeared normal. In this family, a total of six affected patients over three generations showed similar corneal phenotypes; such as onset in the second decade; recurrent corneal erosions with severe corneal ulceration; and the requirement for corneal transplantation with amyloid recurrence following penetrating keratoplasty (pedigree in Figure 1A). Histological examination of corneal buttons from three different individuals in this family also showed similar histopathological features consisting of amyloid deposits (not illustrated). Furthermore, we did not detect an intraepithelial cysts, which are suggestive of EBMD.

#### Family B

**Figure 2:**
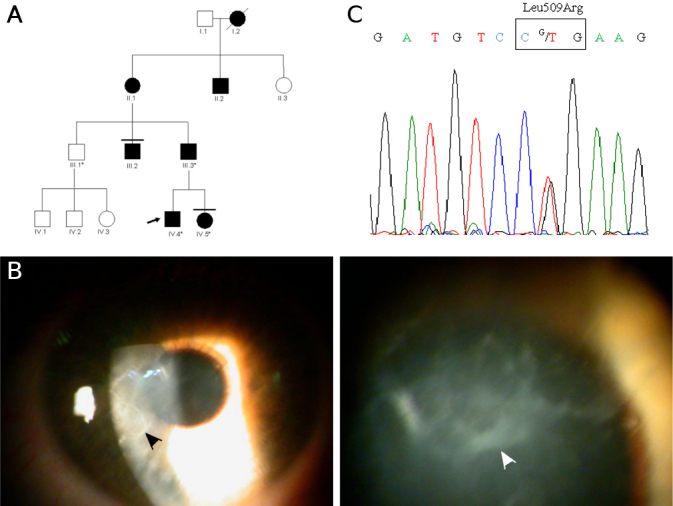
Family B. **A**: Pedigree of this family with an atypical lattice corneal dystrophy. **B**: Slit-lamp examination revealed bilateral map geographic-like opacities in the sub-epithelium with lattice lines (arrowhead) within the corneal stroma in the 41-year-old man (IV.4). **C**: Electropherograms of exon 11 of *TGFBI* gene. The proband IV.4, showing a heterozygous thymine to guanine change at codon 509 (c.1526T>G, p.Leu509Arg).

This family was clinically evaluated in the Department of Ophthalmology at Tours Hospital. The index patient (IV.4) was a 41-year-old man from a French family in which several members, spanning four consecutive generations, were clinically affected with a very similar clinical course and consistently presented with painful corneal erosions with geographic opacities seen at the level of Bowman’s layer (Figure 2A). The patient IV.4 complained, from the age of 25 years, of repetitive episodes of corneal erosions initially only affecting the right eye. The seizure frequency gradually increased, and epithelial erosions progressively affected both eyes and were accompanied by pain and visual deterioration that lead to rapidly penetrating keratoplasty. At this initial stage, the diagnosis was herpes keratitis although laboratory investigations for corneal infection including *Herpesviridae* were always negative. Slit-lamp examination revealed bilateral map geographic-like opacities in the sub-epithelium with lattice lines within the corneal stroma (Figure 2B). To date, no corneal material from this patient or from other affected family members has been available for histological examination.

#### Family C

**Figure 3:**
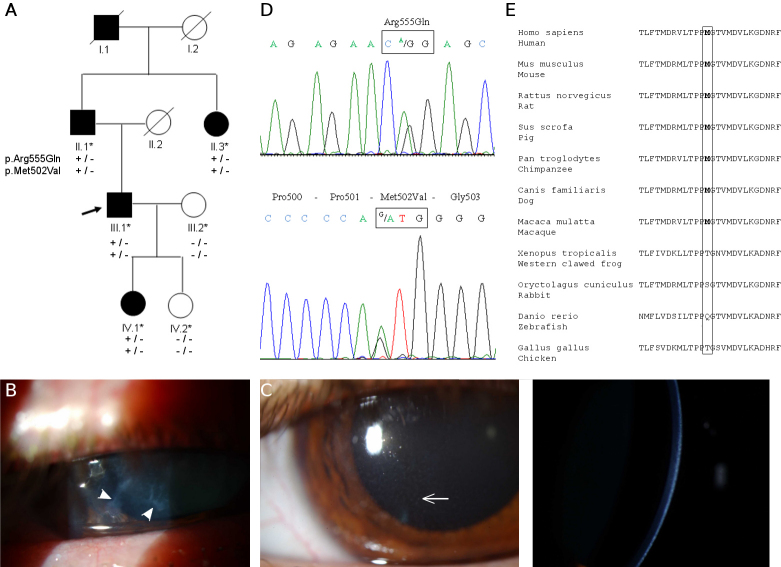
Family C. **A**: Pedigree of the family C included five affected individuals, spanning four generations, with an atypical Thiel-Behnke CD. **B**: The proband (III.1) at 49 years of age; irregular epithelial thickening with multiple subepithelial and stromal dot-shaped non-coalescent opacities are shown (arrowhead). **C**: The affected individual IV.1 at 21 years of age; slit-lamp examination revealed multiple dot-shaped (fine arrow) and diffuse opacities only in the sub-epithelial area. **D**: Electropherograms of exon 11 and 12 of the *TGFBI* gene are shown. The proband IV.1 has a complex allele composed of a novel change p.Met502Val (c.1504A>G in exon 11) and p.Arg555Gln (c.1664G>A in exon 12). **E**: Partial amino acid sequence alignment of keratoepithelin for comparison among eleven species.

This family has been clinically evaluated in the Department of Ophthalmology at Montpellier Hospital. This third French family included five affected individuals over four generations, all of whom presented with a similar corneal disease with autosomal dominant inheritance (Figure 3A). For all affected patients, the onset of the disease began in the first decade of life with unexplained chronic keratitis and conjunctivitis. The patients complained of the sensation of foreign bodies in the eye together with pain, redness, and severe photophobia, and they presented with opacities at the level of Bowman’s layer leading to progressive visual impairment between the 4th and 5th decades of life. At the time of diagnosis, the proband (patient III.1) was a 49-year-old man who had been managed since childhood for unexplained chronic keratitis and conjunctivitis leading to progressive decreased visual acuity (a visual acuity of 1/10 in the right eye and 3/10 in the left eye). A slit lamp examination of both eyes revealed an irregular epithelial thickening with multiple subepithelial and stromal dot-shaped non-coalescent opacities localized to the central cornea (Figure 3B). Lens examination showed a phacosclerosis in both eyes. His 21-year-old daughter (IV.1) exhibited identical findings in both corneas and had also had keratitis and conjunctivitis from the age of 14 months, together with severe photophobia (Figure 3C). Slit-lamp examination revealed multiple dot-shaped and diffuse opacities confined to the sub-epithelial area and without any fluorescein-stained regions (Figure 3C). Family members did not show any clinically evident lattice-type lesions, and the corneal opacities involved only the superficial stromal layers of the cornea. No family member has undergone corneal transplantation, therefore the nature of the deposits associated with the corneal dystrophy could not be determined. The ophthalmologists confirmed a clinical diagnosis of Bowman’s layer CD but its subtype could not be ascertained because the clinical features did not meet the diagnostic classification of either Reis-Bucklers CD or Thiel-Benhke CD. Therefore, this corneal dystrophy did not receive a specific initial clinical diagnosis and was classified as atypical Bowman’s CD. Further, based on the results of the molecular analysis, this corneal dystrophy was reclassified as atypical Thiel-Benhke CD, although the confirmation of the presence of curly fibers on TEM is missing.

#### Case D

**Figure 4:**
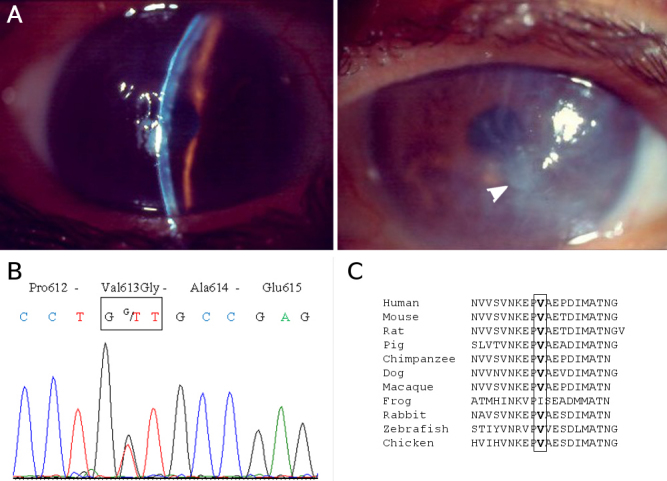
Case D at 80 years of age. A: Representative images of corneal opacities (arrowhead) seen in the right eye of case D, at 80 years of age. **B**: Electropherograms around the codon 613 in exon 14 of the *TGFBI* gene. The patient is heterozygous for a novel mutation, thymine to guanine substitution at position 1828 (c. 1828T>G), causing the p.Val613Gly amino acid exchange. **C**: Partial amino acid sequence alignment of keratoepithelin with ten of its orthologs. The boxes indicate valine at position 613 in the human protein.

The patient was an Algerian male, born in 1925, who was referred, at the age of 80, for evaluation of bilateral corneal dystrophy to the Hotel-Dieu Hospital in Paris (Figure 4A). A corneal graft of the right eye was performed and on histological examination numerous findings suggested a lattice type corneal dystrophy. The majority of the cornea epithelium was abraded; the Bowman's layer was slightly wrinkled at the anterior corneal surface. The stroma appeared slightly thickened and contained eosinophilic deposits. These aggregates did not take up Masson's trichrome stain but showed positive staining with Congo red and thioflavin-T. This patient has 12 children but little clinical information is available on them, except for one child born in 1954, who has been monitored in Algeria and who has exhibited small dots and a star-shaped opacity.

### *TGFBI* sequence analysis and prediction analysis

In Family A, *TGFBI* screening showed a c.1526T>C heterozygous nucleotide substitution in exon 11 that modified the leucine at codon 509 to proline: p.Leu509Pro (Figure 1D). This mutation, which was detected in five affected individuals but not in five healthy family members, suggests that p.Leu509Pro is highly likely to be a disease-causing mutation. Moreover, this *TGFBI* mutation was not detected in the ethnically matched controls tested in our study (see Methods). In Family B, all three affected members (III.3, IV.4, and IV.5) were found to be heterozygous for a c.1526T>G change at exon 11, predicted to result in the substitution of leucine for arginine at codon 509: p.Leu509Arg (Figure 2C). This mutation was not detected in the unaffected individual (III.1), nor in the ethnically matched control individuals. All bioinformatics algorithms showed that leucine 509 is highly conserved across several animal species (e.g., mice, chickens, chimpanzees, and dogs) as well as in the human protein, periostin; predicting that mutations at this position would be expected to adversely affect the function or structure of the encoded protein.

In Family C, *TGFBI* sequence analysis in affected individuals (II.1, II.3, III.1, and IV.1) revealed two heterozygous nucleotide changes that cosegregated with the corneal disease. The first nucleotide variation is a base pair change in exon 11 at nucleotide position 1504 (A→G), which converts a methionine at codon 502 into a valine: p.Met502Val (Figure 3D). The second nucleotide change affects exon 12 of *TGFBI,* and corresponds to the p.Arg555Gln pathogenic mutation classically associated with Thiel-Behnke CD. Family segregation analysis showed that both p.Met502Val and p.Arg555Gln *TGFBI* variants are present in four affected family members (II.1, II.3, III.1, and IV.1) spanning three generations, supporting therefore that these two variants are in allelic association on the same deleterious chromosome (Figure 3A). Mutational screening of the p.Met502Val mutation in our ethnically matched French control population did not detect this variant. In silico analysis predicted the p.Met502Val as being most probably a tolerant variant. We further examined evolutionary conservation of the methionine residue at position 502 using multiple TGFBI protein sequence alignments. The data indicated that Met502 is strictly conserved in mice, rats, pigs, macaques, chimpanzees, and dogs but is replaced by threonine in frogs and chicken, serine in rabbits, or glutamine residue in zebrafish (Figure 3E).

In case D, a novel disease-causing nucleotide change was identified in the heterozygous state at exon 14: c.1828T>G, leading to the substitution of valine for glycine at codon 613 (p.Val613Gly; Figure 4B). Further screening showed two additional variants in the heterozygous state, p.Leu472Leu and c.1804–35C>T, which were previously identified as polymorphisms (rs1133170 and rs45626931, respectively). Unfortunately, family segregation of this novel variant could not be performed since the patient’s family members were not available. However, this *TGFBI* mutation was absent in 200 chromosomes from the ethnically matched Algerian control individuals. The conservation level of residue Val at position 613 is high: it is conserved in domains 3 and 4 of the homologous protein fasciclin I in *Drosophila melanogaster* and is also highly conserved across species (Figure 4C). SIFT, PolyPhen, SNPs3D, and PMut algorithms predicted that the mutation p.Val613Gly is a deleterious substitution causing an alteration in protein structure or function.

### Computational alanine scanning

We performed a computational alanine scan on the keratoepithelin structure using the FoldX 2.5.1 program to examine the importance of Met502, Leu509, Arg555, and Val613 side chains for protein stability. A decrease in the protein stability was observed for Leu509 and Val613 upon mutation to alanine of 3.25 kcal/mol and 1.84 kcal/mol, respectively. This observation confirms the importance of the presence of both residues for the structural stability of keratoepithelin and suggests plausible support for the destabilizing effect of Leu509 and Val613 mutations. In contrast, the mutation to alanine resulted in an increase in structural stability by 0.58 kcal/mol for position 502 and a non-significant decrease of structural stability by 0.3 kcal/mol for position 555. Indeed, both residues, Met502 and Arg555, are solvent-exposed and are therefore not crucial to protein stability.

### Molecular dynamics of wild type keratoepithelin protein and its p.Val613Gly variant

MD simulations in explicit solvent were performed for the wild type NMR structure of keratoepithelin and its p.Val613Gly mutant. The geometry analysis shows that during the 10 ns of MD simulation, the wild type protein was only slightly more stable than the mutant protein. The averaged root mean square deviation (RMSD) from the starting structure, calculated for the backbone atoms, was 1.4 Å for the wild type protein and 1.7 Å for the p.Val613Gly mutant. The folding free energy of 100 simulation frames revealed, however, a significant difference in molecule stability during the simulation. The mean folding free energy value of the wild type keratoepithelin conformation was 3.1 kcal/mol lower than the average value for the Val613Gly mutant, according to FoldX 2.5.1 program calculations.

### NMR structure of the FAS1 domain 4 of keratoepithelin

**Figure 5:**
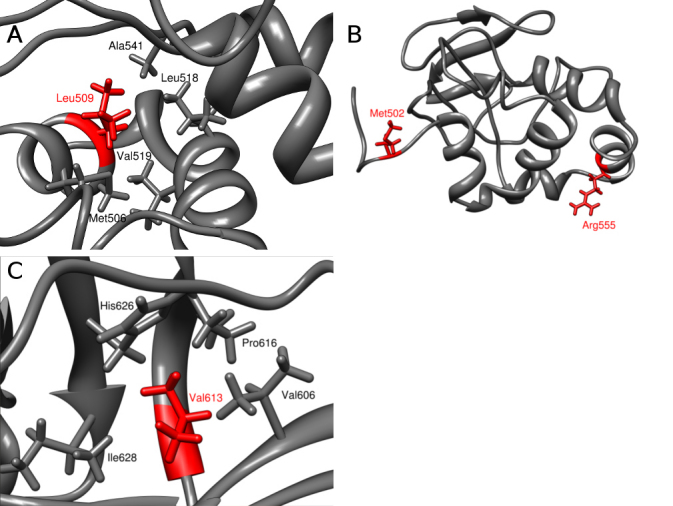
Local structure around the mutated residue in the fourth domain of the keratoepithelin protein (1x3b PDB code). The protein is shown in cartoon representation. Leu509 (**A**), Met502 and Arg555 residues (**B**), Val613 (**C**) and the neighboring residues are shown in stick representation in red and gray, respectively.

According to the NMR structure of the fourth domain FAS1 of keratoepithelin, the Leu509 residue is located in the NH_2_-terminal αL helix. Its side chain points toward the interior of the protein and interacts with the hydrophobic side chains of Ala541 and Val519 (Figure 5A). The other neighboring residues of Leu509 are Val505, Met506, Val508, Leu518, Ile522, and Val625. The aberrant presence of a polar arginine side chain at this position is incompatible with the hydrophobic character of the surrounding residues and with the tight packing of their side chains, hence probably leading to the incorrect folding of this domain. The Met502 is located at the linker region between the FAS1 third and fourth domains and outside the compact protein core (Figure 5B). We presume that the fragment is solvent-exposed and that mutation to residues more hydrophobic than methionine, such as valine, can affect protein folding. Furthermore, the methionine amino acid residue contains a sulfur atom, which is susceptible to oxidation and is slightly more bulky. The residue Arg555 is located at the solvent exposed α4 helix, on the other side of FAS1 domain 4 of the keratoepithelin protein and the potential interaction of Met502Val and Arg555Gln residues can therefore be excluded (Figure 5B). The Val613 residue is situated on the β6 strand and contributes to the hydrophobic core formed by the β5, β7, and β6 strands. The side chain of this residue is in contact with the Val606, Val608, and Pro616 side chains (Figure 5C). This observation suggests that residue Val613 has structural importance. The substitution to glycine at this position creates a cavity within the densely packed hydrophobic interior of the protein, which probably influences protein stability.

## Discussion

Molecular *TGFBI* gene screening of three unrelated French families with autosomal CD and one isolated case of Algerian origin identified four different mutations, including a novel pathogenic variant and a complex *TGFBI* allele. The remaining *TGFBI* variants that we report on correspond to two distinct missense mutations affecting the same residue at position 509 of keratoepithelin: p.Leu509Pro and p.Leu509Arg. We show that both Leu509 variants of *TGFBI* cause corneal dystrophy of the lattice type. These two *TGFBI* variants have previously been described on only one occasion in separate studies and have been associated with different clinical diagnoses [12,13].

### The Leu509 variants

The p.Leu509Pro variant has previously been reported in a German family having a Reis-Bucklers-like CD, which was clinically characterized by opacities with geographic patterns in the subepithelium and the anterior stroma [13]. Although no lattice lines were evident from slit-lamp examination, both amyloid and non-amyloid aggregates were found in the affected corneas in this family. The phenotype in our patients with the p.Leu509Pro variant includes similar opacities that appear geographic in shape in the superficial layer, but which are associated with fine and discrete lattice lines in the deeper stromal layers. Histopathology showed that all deposits were amyloid in nature, and the disease was classified as a lattice CD. The two families previously described as having the p.Leu509Pro variant, exhibited a corneal phenotype characterized by amyloid subepithelial and stromal opacities and inconstant formation of non amyloid deposits. We have no clear explanations for this histopathological variability between the two families, but previous reports also described variability in phenotypes concerning the mutation of p.Gly623Asp, which has been associated with LCD type I/IIIA, Reis-Bucklers CD and atypical CD [18,19]. It remains a possibility that non-amyloid deposits could have been missed in our patients because they could be masked by amyloid aggregates, alternatively they also could occur later in the course of the disease. However, three corneal buttons from different family members of differing ages were examined and all showed similar histopathological features with only amyloid deposits. The p.Leu509Arg variant identified in our second French family is associated with clinical features that are very similar to those presented by our patients suffering with the p.Leu509Pro mutation, including painful corneal erosions in the second decade of life and geographic opacities at the level of Bowman’s membrane with lattice lines in the deeper layers of the stroma. Although deposits of amyloid material could not be demonstrated on histologic examination of diseased corneas with p.Leu509Arg, the presence of lattice lines at the biomicroscopic level is highly suggestive of lattice type corneal dystrophy. This clinical diagnosis therefore differs from that reported by Boutboul et al. for the p.Leu509Arg variant in another French family with EBMD [12]. Ambiguity in the diagnosis of patients with *TGFBI* p.Leu509Arg variant could result from the interpretation of corneal erosions at a stage where lattice lines are not easily detectable during slit-lamp examination. No corneal specimen was available for the family carrying the p.Leu509Arg variant reported by Boutboul et al. [12] to determine whether amyloid aggregates were detected or not. Nine *TGFBI* mutations in this domain are amyloidogenic, and three leucine residues located close to Leu509 (Leu518, Leu527, Leu569), mutated to arginine or proline, have been described in families with various forms of LCDs [20-23]. The structural importance of the Leucine 509 of keratoepithelin is confirmed here by computational alanine scanning data, which revealed that the mutation of this residue to alanine causes a decrease in the stability of more than 3 kcal/mol. In view of the fact that protein stability, measured as the free energy difference between the folded and unfolded states, has a value typically ranging between 5 and 15 kcal/mol for small proteins, the loss of stability is remarkable [24]. This information argues in favor of amyloid formation for mutations affecting leucine 509 residue, and we think that patients with p.Leu509Arg mutation of *TGFBI* should not be classified as EBMD, but rather as having a lattice form of corneal dystrophy. In families A and B, no microcysts in the corneal epithelium were detected. We also investigated a small set of patients who were diagnosed as having EBMD, but in whom no pathogenic mutation in the *TGFBI* gene was found. It could be noted that our patients with both of the Leu509 *TGFBI* mutations had painful and recurrent epithelial erosions that were sometimes complicated by severe corneal ulcers. Although recurrent corneal erosions are a common finding in various forms of *TGFBI* CDs, such atypical and severe clinical presentation, which predominates the clinical picture for several years before the lattice lines become clinically evident, may lead to incorrect diagnoses of EBMD or herpes keratitis being made.

### The p.Val613Gly variant

We have identified a novel mutation, p.Val613Gly, associated with a corneal dystrophy of the lattice type. Although familial segregation of p.Val613Gly could not be assessed, different findings support that p.Val613Gly is pathogenic. With respect to the structure of keratoepithelin, we showed that the Val613 residue is of structural importance. The computational alanine scanning and the molecular dynamics simulation of the p.Val613Gly mutant compared to the wild type protein show a decrease in the protein stability compatible for protein aggregation, which is the physiopathology underlying molecular mechanism that characterize CDs linked to *TGFBI*. The mutation to glycine might also lead to improper folding in view of the fact that the glycine backbone structure is not restricted by the presence of a side chain and can adopt any conformation which might lead to misfolding. The p.Val613Gly mutation may also facilitate the β-pleated sheet structure favoring the accumulation of insoluble protein material and amyloid formation. In agreement with the predicted dramatic effect of the p.Val613Gly mutation, is the presence of amyloid deposits from corneas of case D.

### [p.Met502Val;p.Arg555Gln] variants

In Family C, we have identified a complex TGFBI allele [p.Met502Val;p.Arg555Gln] associated with a corneal dystrophy of an unspecified type. These two heterozygous nucleotide changes in exon 11 and exon 12 are present in all affected family members and are predicted to change two residues in the FAS1 domain 4 of the keratoepithelin, where the *TGFBI* disease-causing mutations are clustered. The status of the p.Met502Val variant is questionable in terms of its ability to determine whether it is a neutral or an intolerant variant. Numerous points argue in favor of its pathogenicity. First, in 2009, Zenteno et al. [25] identified the p.Met502Val variant as the disease-causing mutation in a Mexican family. Second, this variant was exclusively found in affected patients and was completely absent from ethnic Mexican and French control subjects. Additionally, p.Met502Val variant has not been registered in the Single Nucleotide Polymorphism (SNP) database. Third, we note that even if the degree of conservation of Met502 among TGFBI orthologs is moderate, a valine residue at this amino acid position is never found in any species. Fourth, the p.Met502Val variant is predicted to affect the folding of keratoepithelin. Fifth, this region of TGFBI has been previously associated with numerous disease-causing mutations [12,13,26,27]. Based on all of these assessments, the p.Met502Val variant is highly likely to be a disease-causing mutation. However, two major unresolved questions remain to be elucidated. First, it would be of importance to define the characteristic features of corneal deposits associated with the p.Met502Val variant, and second, to determine the potential phenotypic modifier effect of the p.Met502Val variant over the p.Arg555Gln variant. Indeed, we have previously shown that the association in *cis* of both the p.R124L and delT125-E126 variants gives rise to a specific granular corneal phenotype, resulting most probably from the effect of each molecular defect [28].

In conclusion, this study highlights how phenotype-genotype correlations in *TGFBI* CDs may be particularly complex, making the availability of combined histopathological and genetic data necessary for the better classification of these corneal dystrophies. Furthermore, identification of the p.Leu509Arg in a French family with lattice-type corneal dystrophy raises the need to reconsider the role of *TGFBI* in the molecular diagnosis of Epithelial basement membrane dystrophy (EBMD).
